# Supplementary material for: Changes in the Expression of Mitochondrial Morphology-Related Genes during the Differentiation of Murine Embryonic Stem Cells
Source: Stem Cells Int. 2020 Jan 28;2020:9369268. doi: 10.1155/2020/9369268 (PMC7204333; doi:10.1155/2020/9369268)
Supplement: Supplementary Materials — Supplementary Figure 1: expression pattern of TOM20 on days 0, 3, 6, 9, 12, and 15 after differentiation of ESCs. Green dots represent mitochondria. Nuclei was counterstained with DAPI. Scale bars = 10 μm. Supplementary Figure 2: (a) the mitochondrial perimeter (μm) in ESCs on differentiating days 0, 3, 6, 9, 12, and 15. (b) The mitochondrial area (μm2) in ESCs on differentiating days 0, 3, 6, 9, 12, and 15. Data are presented as mean ± SEM for n = 50 independent experiments. ∗p < 0.05, ∗∗p < 0.01, and ∗∗∗p < 0.001 versus D0. Supplementary Figure 3: (a) correlation analysis between the Mfn2/Dnm1L ratio and the maximal length of mitochondria normalized to ESCs (D0). (b) Correlation analysis between the Mfn1/Fis1 ratio and the maximal length of mitochondria normalized to ESCs (D0). Supplementary Figure 4: (a) normalized protein level of DNM1L protein on days 0, 3, 6, 9, 12, and 15 after differentiation of ESCs. (b) Normalized protein level of MFN2 protein on days 0, 3, 6, 9, 12, and 15 after differentiation of ESCs. Protein expression levels were normalized to those of Actb. All data are presented as mean ± SEM for n = 3 independent experiments. ∗∗∗p < 0.001 versus D0. [file 9369268.f1.docx]

Supplementary information


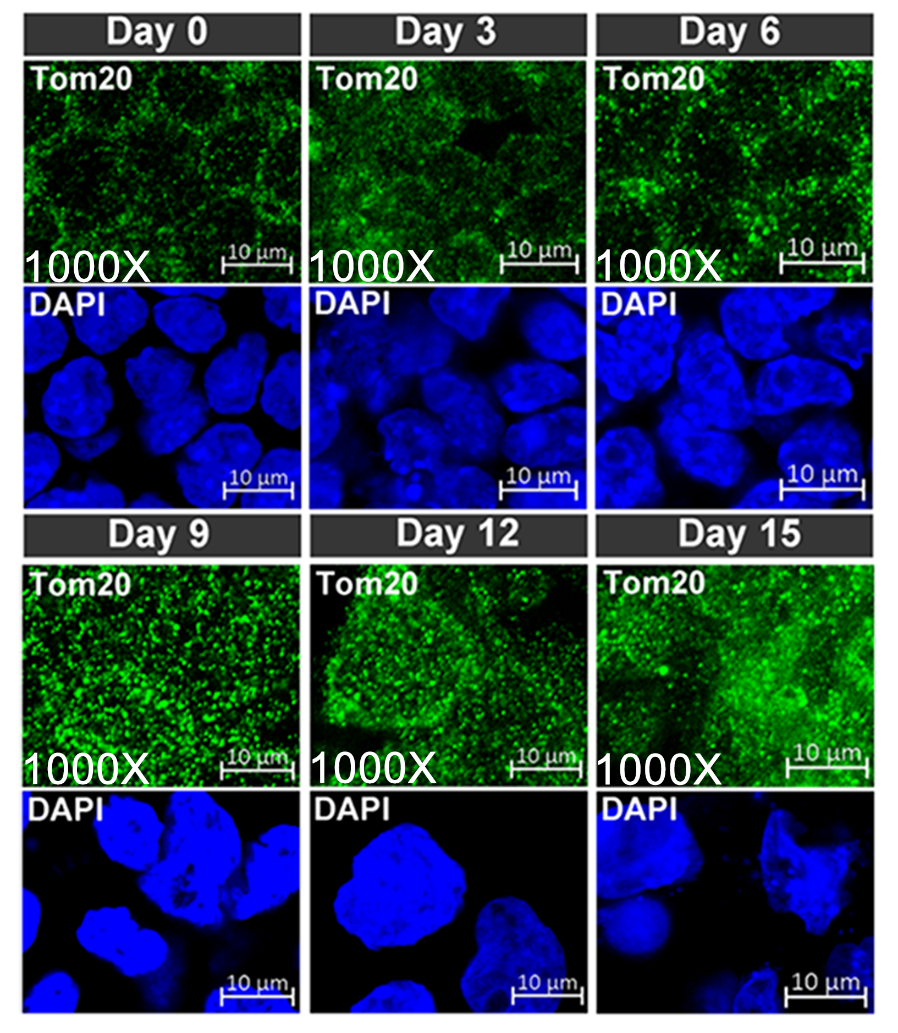


**Supplementary Figure 1.** Expression pattern of TOM20 on days 0, 3, 6, 9, 12, and 15 after differentiation of ESCs. Green dots represent mitochondria. Nuclear was counterstained with DAPI. Scale bars = 10 µm.


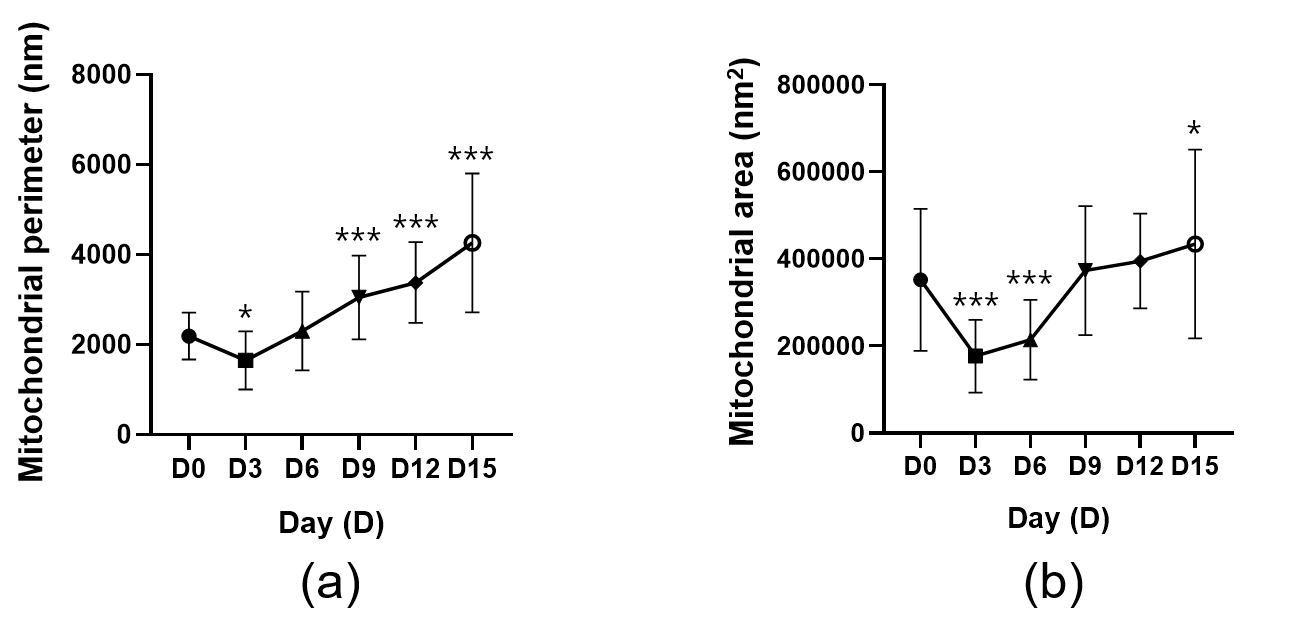
**Supplementary Figure 2.** (a) The mitochondrial perimeter (nm) in ESCs on differentiating days 0, 3, 6, 9, 12 and 15. (b) The mitochondrial area (nm^2^) in ESCs on differentiating days 0, 3, 6, 9, 12 and 15 (quantified n=128 from 21 cells of day 0, 28 cells of day 3, 16 cells of day 6, 15 cells of day 9, 15 cells of day 12, and 17 cells of day 15). Data are presented as mean ± SEM for an experiment. *p < 0.05 and ***p < 0.001 versus D0.


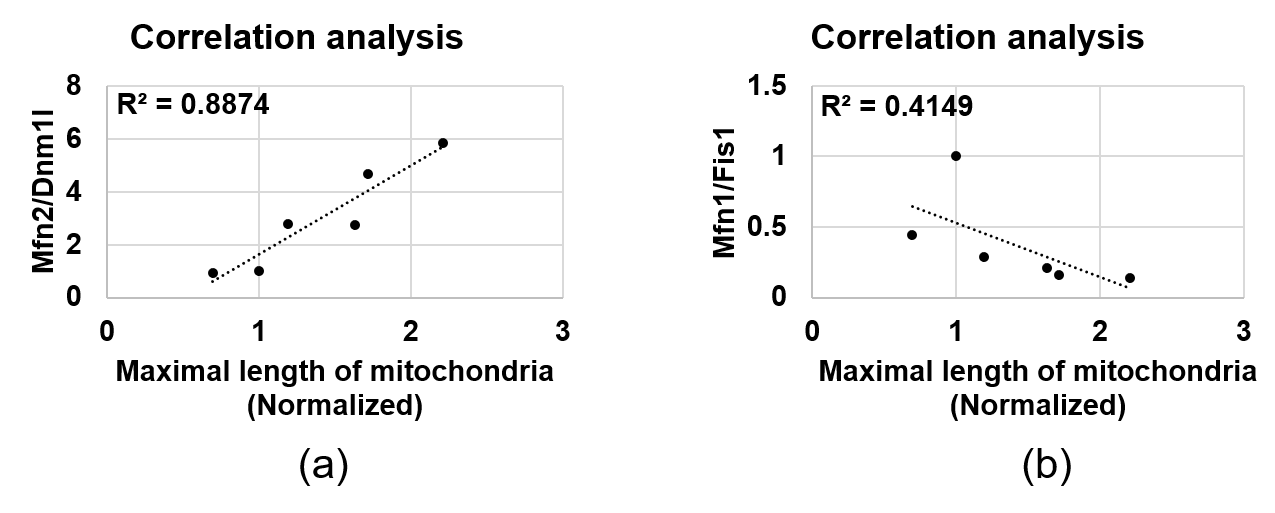


**Supplementary Figure 3.** (a) Correlation analysis between Mfn2/Dnm1l ratio and the Maximal length of mitochondria normalized to ESCs (D0). (b) Correlation analysis between Mfn1/Fis1 ratio and the Maximal length of mitochondria normalized to ESCs (D0).


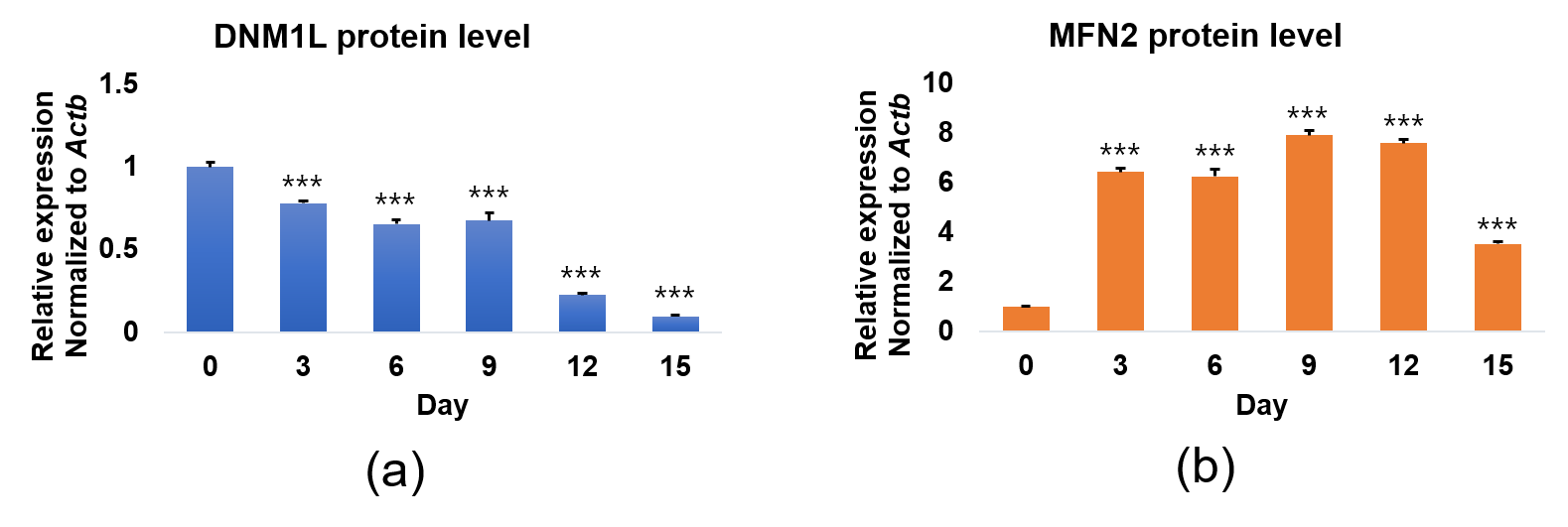


**Supplementary Figure 4.** (a) Normalized protein level of DNM1L protein on days 0, 3, 6, 9, 12, and 15 after differentiation of ESCs. (b) Normalized protein level of MFN2 protein on days 0, 3, 6, 9, 12, and 15 after differentiation of ESCs. Protein expression levels were normalized to those of *Actb*. All data are presented as mean ± SEM for n = 3 independent experiments. ***p < 0.001 versus D0.
